# Uncovering the ecology of clinical education: a dramaturgical study of informal learning in clinical teams

**DOI:** 10.1007/s10459-020-09993-8

**Published:** 2020-09-20

**Authors:** Peter Cantillon, Willem De Grave, Tim Dornan

**Affiliations:** 1 Discipline of General Practice, National University of Ireland, Galway, Republic of Ireland; 2 School of Health Professions Education, Maastricht University, Maastricht, The Netherlands; 3 School of Medicine, Dentistry and Biomedical Sciences, Queens University Belfast, Belfast, UK

**Keywords:** Asymmetries of power, Clinical education, Ethnography, Implicit curriculum, Impression management

## Abstract

Off-the-job faculty development for clinical teachers has been blighted by poor attendance, unsatisfactory sustainability, and weak impact. The faculty development literature has attributed these problems to the marginalisation of the clinical teacher role in host institutions. By focusing on macro-organisational factors, faculty development is ignoring the how clinical teachers are shaped by their everyday participation in micro-organisations such as clinical teams. We set out to explore how the roles of clinical teacher and graduate learner are co-constructed in the context of everyday work in clinical teams. Using an ethnographic study design we carried out marginal participant observation of four different hospital clinical teams. We assembled a dataset comprising field notes, participant interviews, images, and video, which captured day-to-day working and learning encounters between team members. We applied the dramaturgical sensitising concepts of impression management and face work to a thematic analysis of the dataset. We found that learning in clinical teams was largely informal. Clinical teachers modelled, but rarely articulated, an implicit curriculum of norms, standards and expectations. Trainees sought to establish legitimacy and credibility for themselves by creating impressions of being able to recognise and reproduce lead clinicians’ standards. Teachers and trainees colluded in using face work strategies to sustain favourable impressions but, in so doing, diminished learning opportunities and undermined educational dialogue. These finding suggest that there is a complex interrelationship between membership of clinical teams and clinical learning. The implication for faculty development is that it needs to move beyond its current emphasis on the structuring effects of institutional context to a deeper consideration of how teacher and learner roles are co-constructed in clinical teams.

## Introduction

There has been growing disquiet about the quality of graduate clinical education (Irby 1995; Parsell 2001; Spencer 2003; Kennedy et al. 2005; Swanwick 2008; Norman 2012; Steven et al. 2014; Wiese et al. 2018). At first, the concerns related to its perceived messy and unsystematic nature (Irby 1995; Spencer 2003; Parsell 2001). The advent of working time directives led to apprehensions about the fractured and truncated clinical education experience of graduate trainees (Vanstone et al. 2014; Wiese et al. 2018). More recently, clinical education has been characterised as inefficient (Bolster and Rourke 2015) and ineffective due to tensions between education, the safe provision of clinical services, and research (Kennedy et al. 2009a; Goldszmidt et al. 2014; Patel et al. 2018; Wiese et al. 2018). These shortcomings have been further highlighted by the widespread adoption of competency-based curriculums in graduate medical education (Ten Cate and Billett 2014). The perceived inefficiencies of graduate clinical education have led to increased calls for action, driven largely by enhanced scrutiny of clinical education by professional training and accreditation bodies (Dornan et al. 2019).

Formal faculty development and credentialing of clinical teachers was put forward as a solution to the perceived problems of clinical education (McLeod et al. 2003; Steinert et al. 2006, 2009, 2019; Swanwick et al. 2010; O’Sullivan and Irby 2011; O’Sullivan et al. 2014). In the event, faculty development initiatives were poorly attended and had disappointingly little impact (Stone et al. 2002; Starr et al. 2003; Steinert et al. 2009; Hafler et al. 2011). The problems encountered were attributed to cultural factors that made institutions, (and therefore clinical teachers) resistant to the influence of faculty development (Hafler et al. 2011; Graham and Dornan 2013). The faculty development community, now more aware that organisational contexts shape teacher development, has shifted its scholarly emphasis. Rather than achieving impact by providing off-site teacher training, it is increasingly focusing its attention on relationships between faculty development and the institutional contexts in which clinical teachers work (Steinert 2010; O’Sullivan and Irby 2011; Hartford et al. 2017; Sheehan et al. 2017; van Lankveld et al. 2017a; Bates et al. 2018). This relatively recent contextual turn has focused mainly on ‘macro-organisational’ contexts such as the relationship between institutional norms and teacher development. Far less attention has been paid to how clinicians become teachers in the fluid ‘micro-organisational’ contexts of clinical teams (Cantillon et al. 2016). Clinical teams are important contexts because it is by participating as members of teams that graduate trainees learn clinical practice. We therefore set out to explore how the role of clinical teacher and graduate learner are co-constructed in the context of everyday working relationships within hospital based clinical teams.

Researching clinical education is challenging because work and learning are so enmeshed that they are barely distinguishable from one another (Steinert et al. 2017). Most clinical learning is informal and situated in the hierarchical structures of practising teams. (Hibbert et al. 2018). Informal learning is an ad hoc and implicit process, in which graduate trainees become legitimate team members by developing the non-canonical capability of looking, talking and behaving like a doctor (Hoffman and Donaldson 2004; Monrouxe et al. 2009). They do this by mastering teams’ ‘implicit curriculums’ of shared practices, rules of thumb and embodied understandings (Hindmarsh and Pilnick 2002; Balmer et al. 2009; Hägg-Martinell et al. 2015). The majority of this is so well hidden, even from the view of teachers and learners, that self-report studies have revealed little about the realpolitik of learning in clinical teams (Lingard et al. 2012; Paradis et al. 2015).

Observational studies have proved more useful (Lewin and Reeves 2011; Leslie et al. 2014). Ethnographic research has shown that graduate trainees, who are motivated to “talk the talk” and master “the rules of the game“, will adapt particular ways of seeking help or asking questions in order to be recognised as legitimate by their supervisors (Bleakley 2002; Hawryluck et al. 2002; Lingard et al. 2002a, b; Kennedy and Lingard 2007; Kennedy et al. 2009b; Cristancho et al. 2013; Ott et al. 2018). Other informative studies have looked at how clinical supervisors grant graduate trainees an appropriate degree of autonomy whilst assuring patient safety (Kennedy et al. 2007; Goldszmidt et al. 2012, 2015). There has been less research into how the roles of teacher and learner are shaped within clinical teams. The limited research that does exist has shown that team members learn and reproduce implicit choreographies of behaviour during bedside clinical encounters (Balmer et al. 2012). Whilst helpful in revealing some aspects of the politics of clinical education, these studies do not show how teachers and graduate trainees co-construct their roles. The aim of this study was to contribute to the scholarship of faculty development by observing the social production of clinical education practice in the context of hospital-based clinical teams.

### Theoretical framework

Symbolic interactionism, particularly Goffman’s dramaturgical metaphor of the stage, provides a useful framework for exploring how people (co-)construct roles for themselves and others during social interactions (Goffman 1959). According to this theory, Goffman showed how individuals seek to present the best possible impression of themselves to others through processes of impression management, governed by implicit social rules. Goffman coined the term “face” to describe how people present themselves to others in accordance with anticipated social expectations (Goffman 1967). He used the term “face work” to refer to how actors collaborate to maintain positive face, suppress discrepant impressions, and avoid embarrassment (Goffman 1967). To do this, actors have to share tacit knowledge about the nature and purposes of their performance. Teams, from this perspective, are societies of insiders, “in the know”, collaboratively giving audiences chosen impressions. Team members are “reciprocally dependent” on one another because any one of them could use their insider knowledge to give the show away (Goffman 1967). The peculiar nature of clinical teams make them particularly interesting in this regard. Whilst patients experience only the performance of clinical care, embedded within this clinical action is the hierarchical performance of clinical education. It is probably this complex relationship that diminishes the effectiveness of off-the-job faculty development. Dramaturgy is therefore, a very suitable theoretical lens to interpret the normative effects of language and behaviour on the co-construction between senior clinicians and graduate trainees of professional identity in clinical workplaces (Ellingson 2005). To achieve our aim, this research uses the sensitising concepts of impression management and face work to structure an analysis of how senior clinicians and graduate trainees in a sample of hospital-based clinical teams co-produce the roles of teachers and learners within practice .

## Methods

Ethnography is ideally suited for studying complex social and cultural phenomena such as clinical education in the context of hospital teams (Warmington and McColl 2017; Balmer et al. 2018; Ott et al. 2018). Prolonged observation of practice in natural settings, combined with participant interviews, can reveal taken-for-granted practices, perspectives and cultural features that are not apparent in self-report qualitative designs (Reeves et al. 2013; Leslie et al. 2014). By assembling multiple forms of data from a variety of sources, ethnographic accounts can provide a deeper and more comprehensive understanding of the phenomenon under investigation (Leslie et al. 2014). We therefore used the well-established ethnographic approach of marginal participant observation (Hammersley and Atkinson 2007) to guide our data collection. We interpreted the data using thematic analysis (Creswell 2014) informed by the dramaturgical concepts of impression management and face work (Goffman 1959).

### Setting

The settings for this research were medical and surgical teams in two teaching hospitals in Ireland. Whilst Irish hospital care is acknowledged to be of a generally high standard (Irish Health Insurance Authority 2018), it faces considerable challenges in achieving those standards because of high bed occupancy rates, (often close to 100%), a relative undersupply of diagnostic resources and insufficient community healthcare structures to facilitate timely patient discharges. These factors combine to create a pressurised environment, where clinical teams are often frustrated in their efforts to get hospital beds for new patient admissions, experience delays in getting timely access to key services, and have to defer patient discharges back into the community.

The clinical teams recruited for this research were typical of Irish hospital practice in that they were led by one or more consultant specialists, (attendings) and included 4–6 graduates in various training grades. The graduate trainees typically ranged in seniority from registrars (senior residents) to senior house officers, (SHOs; junior residents) and interns, (1st year of graduate training). Typically in Ireland, graduate trainees are assigned to a sequence of specialist clinical teams for periods of between 3 and 12 months, each within a designated training programme. Irish hospital clinical teams are hierarchical social structures in which lead clinicians have considerable powers of patronage as well as clinical and educational oversight. The ability of graduate trainees to negotiate legitimacy, autonomy and advancement is under the direct control of senior team members.

### Sample and context

We selected two participating teaching hospitals on the basis that they were associated with different medical schools in adjacent cities in Ireland. We purposively selected one surgical and one medical team in each hospital to represent the differing traditions of internal medical and surgical training. The hospitals selected were both medium-sized, (400–600 beds). Each of the recruited clinical teams was led by a consultant and included 4–6 graduate trainees ranging in seniority from Interns and Senior House Officers, to Registrars.

Prior to recruitment, a gatekeeper in each hospital distributed an information leaflet about the study. The lead author, (PC) then presented information about the study at medical and surgical grand rounds. Clinical teams were selected in the order they volunteered to participate.

### Data collection

Having obtained informed written consent from all team members, PC embedded himself as a marginal participant observer with each participating team for a period of between 12 and 16 weeks, (approximately 10 h or 2 days per week) from late 2015 to 2017. He observed all the team’s activities including ward rounds, outpatients, corridor conversations, meetings, (specialty and multidisciplinary), grand rounds, and social interactions, (e.g. coffee breaks). He recorded his observations using contemporaneous notes that were written up as fully developed field notes following each period of observation (Hammersley and Atkinson 2007; van Maanen 2011). He validated field note observations and insights using informal on-site interviews. He also conducted an exit semi-structured interview with each team member to explore questions and observations derived from the field notes. PC collected video recordings of team interactions in outpatient clinics and captured photographic images of team activities in a variety of clinical settings. He digitally recorded interviews and transcribed both them and representative samples of video recordings. He removed all personal identifiers from field note, video, and interview transcripts to ensure confidentiality and any photographic images used in the analytical process were pixelated.

### Data selection

The data that informed this paper was derived primarily from the field note and interview transcripts, because they provided the richest dataset for capturing the practices of impression management and face work as they occurred in the context of everyday clinical work. Video recordings consisted for the most part of doctor–patient interactions with brief doctor to doctor case presentation interludes. Images captured group configurations and highlighted the part played by objects in constructing practice. As such, video and image data were more revealing about sociomaterial and technical aspects of clinical practice and will be used in a forthcoming companion paper that explores how clinical teacher identity is figured in relation to the cultural worlds of internal medicine and surgery. For the purposes of this paper, video and image data were used for data triangulation purposes, to corroborate or refute emergent insights from field note and interview data analysis.

### Data analysis

A total of 640 h of observation, 34 exit interviews, and 30 h of video recording were assembled over the period of the study. We employed NVivo 12 software, (QSR International 1999) to manage and cross-reference the dataset. We conducted data collection and interpretation iteratively. In practice, this meant that observational field notes and exit interviews were informed by questions emerging from data interpretation and the interpretation process was shaped in turn by dilemmas and patterns identified during data collection, (see example in “Appendix 1''). Sensitised by the concepts of impression management and face work, we used Creswell’s system of thematic analysis to make sense of field note, interview and video transcript data. We identified significant narratives, statements and quotations, and then assembled these into clusters of meaning. We used reflexive group discussion to develop initial ideas and insights into themes. We subjected emergent themes and patterns to further refinement in the light of subsequent field note and interview accounts. As stable themes emerged from the analysis of text, we compared these to evidence of similar practices captured on images and video recordings. Participants in the four clinical teams ‘member-checked’ our interpretation during both the data collection and analysis phases. Once we had derived a stable thematic framework, we again validated our insights with members of participating teams.

### Rigour and reflexivity

Two forms of triangulation—researcher and data triangulation (Flick 1992)—supported the rigour and trustworthiness of our interpretation. The three authors interpreted the data from different disciplinary viewpoints; we tested inferences from one source—e.g. field notes—against observations from other sources—e.g. interviews, and we member checked observations with participants.

In accordance with ethnographic research practice, we used our different subject positions i.e. insider (a general practitioner PC and a hospital specialist TD) and outsider, (an educational psychologist WdG) to provide both emic and etic interpretations of the observed learning environments and thus ensured that self-evident aspects of clinical workplaces were noticed and included in the analysis, (see example in ``Appendix 2''). PC also kept a reflexive diary to ensure that the relationship between his personal perspectives, observations, and interpretations were recorded and transparent.

### Ethics

The research design was approved by the research ethics committees of the two participating teaching hospitals. All participants received information sheet about the research and provided written consent for participation in the research. Any patients or healthcare staff who were incidentally included in video or image data also received an information sheet with researchers’ contact details, which they were asked to read before giving verbal consent to be included in the dataset. If such consent was not forthcoming, we verifiably deleted the relevant data segments.

## Results

Informal learning in all four of the clinical teams that we observed was shaped by an implicit curriculum of establishing two related, but distinct individual impressions: the impression of being a *capable team player* at interfaces between the team and the hospital and the impression of being a *capable team member* at interfaces within the team. To be perceived as a *capable team player*, the graduate learner had to contribute to the team’s self-presentation as an effective and efficient unit at important external interfaces, such as with patients, their families, and in professional set piece events e.g. grand rounds. To be viewed as a *capable team member*, the graduate learner had to demonstrate an ability to align him/herself with the implicit standards and expectations of the lead clinicians. Lead clinicians communicated their implicit standards and expectations using strategies of modelling and embodiment. Learners endeavoured to create positive impressions of themselves as capable team members by recognising, interpreting, and reproducing lead clinicians’ implicit curriculums of standards and expectations. We found remarkable consistency in the impression management practices of the four clinical teams that we observed. The differing contexts of internal medicine and surgery meant that there were differences in the content of the impressions made, but the processes of impression management were consistent across all four teams.

Given that this paper is about the co-construction of teacher and learner roles within clinical teams, the results that follow will for the most part report the co-production between lead clinicians and graduate learners of the impression of being a capable team member. Many of the citations that we use are derived from junior graduate learners, particularly interns, which is very much in keeping with the ethnographic principal that it is the newest members of a social group who are the most aware of its particularities and idiosyncrasies (Hammersley and Atkinson 2007; Leslie et al. 2014; Bates and Ellaway 2016). We have labelled citations indicating participants’ status, their affiliation to an internal medicine team (IM) or a surgical team (S); the team’s location in hospital 1 (H1) or hospital 2 (H2) and the source of the citation i.e. field note or interview.

### The capable team player impression

New entrants to teams learned that a key part of their role was to present the team as efficient and effective.

*On a team it’s trying to get things done as smoothly as possible; that’s the game.* Intern interview IM H1.

Efficiency meant being proficient at negotiating preferential access to valued social goods such as diagnostic resources, hospital beds, and theatre space for the team’s own patients, in competition with other teams. This impression was threatened by the team having an excessive patient census or workload. Being an effective team player, then, meant learning how to resist other teams’ attempts to transfer patients or requests for ‘consults’ (i.e. requests to give specialist opinions on patients under the care of other teams). Considerable social value was attached to being persuasive and assertive at the interface between one’s own team and other teams/hospital services. One intern spoke about learning to negotiate preferential access to radiology on behalf of his team:

*Day one you arrive in and you think “oh the scan will just get done” but you learn you have to play the game ..., show your ace card... try and get them* [your patients] *as high up the list as you can, try and convince them* [the radiology department] *that “oh this needs to be done”. You learn what they want to hear; what will ring alarm bells in their heads..... Nothing is ever clear cut and you have to manipulate things a certain way to get people to listen to you, and get things done…that’s your job.* Intern interview S H2.

Similarly, in hospital contexts where patient beds were hard to find and access to diagnostic services could be delayed, team members learned how to put up pre-requisite barriers to requests and demands from other teams.

*When you ring* [for a consult] *you get met with hostility...People don’t want to be accepting new jobs because if they do that, they’ll just get loads of work. So they try and put up a shield to filter out anything they don’t see as theirs.* Intern interview S H1.

### The capable team member impression: lead clinician’s implicit standards

Whilst each clinical team that we observed had an implicit curriculum of norms, standards, and expectations, these were rarely articulated. Rather, lead clinicians and senior team members tended to model or embody standards in the ways they acted and talked; for example, a change of lead consultant on one of the participating medical teams led to a shift in the team’s implicit standards:

*It is immediately obvious that this is a very different ward round to those that I have witnessed before. We’re walking more quickly. A lot of business talk is done as we move along the corridor. The consultant knows many of the patients that he is going to see and has considerable background knowledge that he shares as he walks. He expects his team to have answers to his questions and he seems a little impatient at times.* Ward Round Field Note IM H1.

One of the junior team members interpreted this shift in the team’s implicit curriculum as follows:

*He has very high expectations. I think that is really good because then you have to try and live up to them...it drives you on to try and learn more...and you learn so much from him. He goes through all the bloods, he goes through all the scans, and as he goes through the scans he’ll tell you what he’s finding on them, and it is always a good way to learn.* SHO interview IM H1.

Based on exit interviews with lead clinicians, we found three domains of team member capability that consultants expected and looked for in their junior staff (See Table 1). These included cognitive capability, (e.g. good memory); narrative capability, (e.g. succinct case presentations); and interpersonal capability, (e.g. knowing one’s place). Whilst never explicitly stated during team interactions, these capabilities informed how senior clinicians judged their juniors and appraised them in terms of readiness for greater autonomy.

**Table 1:** Three domains of team member capability: (the lead clinician perspective)

Capability domains	Lead clinician’s unstated expectations of their team members	Examples from interview data
Cognitive capability	Displays a reliable memory for patients’ details, investigation results, therapeutic progress et cetera. Displays an ability to distinguish effectively between patients with similar presenting conditions Displays an ability to anticipate patient or team requirements—i.e. an ability to recognise and act upon the trajectory of current thinking or actions.	*On the wards its efficiency. There are clinical components obviously to in-patient work, but there is also an ability to differentiate between patients and to know results off the top of your head. Like what’s the CRP? And what are the issues with this man again? ... Memory is hugely important... You can tell that some SHOs; they just blur one patient into another, whereas the better, the more effective doctors are able to distinguish between them.* Consultant interview IM H1
Narrative capability	Displays an ability to encapsulate the patient story, the investigative and physical findings in a succinct case presentation Displays an ability to communicate, (sell) an interpretation of the patient presentation, as well as an investigative and management strategy in a confident and persuasive manner	*That is how you get the measure of people; their knowledge and their ability to communicate clinical cases will inevitably inform the level of surgery that they are going to be doing, assisting or allowed to do. All surgeons will use the phrases common sense and cop-on. That really is the way that it’s judged. By the time you get to SHO, junior registrar* [junior resident] *level you need to have figured out what the important stuff is. You need to filter out the noise and get down to what the important things are.* Senior registrar Interview S H2
Interpersonal capability	Displays “enthusiasm” for learning and involvement, i.e. “showing that you want it” Knows his/her place i.e. knows when to speak up; when to ask and when to stay silent Looks and sounds like he/she knows what is going on or planned; i.e. displays being “in the know”	*I remember learning bronchoscopy, and being there, seeing a couple, and then doing a couple. I was thinking “can I do one now?” I want to do one, let me do one” If they* [trainees] *are not bringing themselves forward and going “can I do this one” you’ll think “well ok, you’re obviously not interested in this aspect of respiratory practice – you can assist”. They have to be showing interest; they have to show that they want it.* Consultant interview. IM H1

### The capable team member impression: the junior doctor performance

Junior doctors endeavoured to recognise and reproduce the unspoken expectations of their seniors. Successful displays of being a capable team member enhanced juniors’ credibility and led to opportunities for practice and greater clinical autonomy. One junior doctor described realising the implicit curriculum within clinical teams as a process of pattern recognition:

*I think a major thing is to cop on and know what’s expected of you. As an SHO it’s about impressions and keeping the boss happy. And the way you keep the boss happy is by looking at what they like and what their patterns are with things, and just following that pattern. I think so much in medicine is just pattern recognition with the team that you’re in.* SHO Interview S H2.

“The “cop on” mentioned by the SHO above was to recognise and reproduce the implicit standards of senior clinicians as outlined in Table 1. Junior doctors used a variety of strategies to create impressions of meeting the implicit expectations and standards of senior team members. Table 2 provides examples of these strategies and their associated social goals.

**Table 2:** Trainee’s displays of being a capable team member

Domains of capability	Making impressions	Indications of social goals
Cognitive (Anticipation/trajectory recognition example)	[Demonstrating anticipatory capability] *It’s about trying to figure out what else might need to be done that hasn’t been asked for yet, like enquire about the patient’s social status, or to anticipate if they are going to need a consult down the line.* SHO Interview IM H2	Displays of anticipation helped to secure greater recognition and autonomy within the team as well as opportunities for practice. *If you arrived on the job and just sat back; held the camera or delayed cutting stitches during operations you would never be allowed to do anything. It’s about being engaged…being interested. That’s the only way that you’ll get trained; you can’t just turn up.* Surgical Registrar Interview S H1
Narrative (Case framing example)	[A good presentation] *shows that you are assertive and you know that you’ve got a management plan in your head and you are sure about what’s good for the patient. To get the consultant on your side, it’s important to have a bit of confidence...* Intern Interview S H2	Displays of narrative capability represented opportunities to frame the team’s conceptualisation of patient cases as well as readiness for independent practice. *They know what the consultant wants to hear. They know what the consultant is going to ask them for. They have it in their minds that there is something going on here. They want to convey this to the consultant and get them onto the same thought process.* Intern Interview IM H2
Interpersonal (Being “in the know” and “knowing one’s place” example)	[The “in the know “assumption] *The consultant requested a nephrology consult prior to discharging the patient. There was no explanation as to why the consult was necessary. I wonder to myself how the SHO and intern know what it is that they are seeking in terms of opinion or recommendations from the nephrology team. It is as if they are implicitly expected to understand the consultant’s thinking and to interpret his requests accurately without checking and reviewing his meaning.* Ward Round Field Note IM H1	Displays of being “in the know” indicated currency of knowledge and awareness. Displays of “knowing one’s place” indicated awareness of hierarchy and self-reliance. *Sometimes you mightn’t be fully clear on why you are getting a consult. You have to read the whole chart trying to figure out why. You can go back to them* [the senior team members]; *they can be quite angry that you are asking. Usually they wouldn’t say anything, but they would be somewhat disgusted...* Intern interview S H2

### Maintaining impressions of team member capability: the face-work collusion

Lead clinicians and trainees employed face work to sustain the impressions that they were presenting and to avoid embarrassment. For example, asking or answering questions brought the potential for reputational damage. Junior doctors learned to be circumspect about who they asked questions of:

*I suppose interns might be reluctant to ask questions of seniors for fear of looking incompetent. Interns would feel reluctant to ask certain questions if they felt SHOs were there: “how will this person see me if I ask this question?” The other interns are your peers and you wouldn’t be as afraid to ask* [them] *a stupid question*. Intern Interview IM H2.

Similarly, junior doctors employed self-restraint in speaking up or asking questions in order to mitigate any risk of loss of face in team settings:

*I think that’s just a fear of being wrong... I never have been one to speak out. Nobody ever volunteers unless they are specifically asked. I guess as you get older, if it is something simple, then that would be embarrassing to miss it on say an x-ray. I think especially with Dr X* [the lead consultant], *his expectations are high, and you want him to think that you are a good doctor, and so I just wouldn’t speak out in case I was wrong.* SHO interview IM H2.

For their part, clinical teachers/supervisors colluded in maintaining junior doctors’ impressions of capability, by avoiding actions that might embarrass the junior team member. For example, clinical teachers/supervisors employed different rules of question etiquette depending on whether they were dealing with medical students or junior doctors:

*I don’t want to challenge them* [the junior doctors]. *I can ask a medical student about antibiotics and so on, but with the team, it’s trickier. They are challenged by the work load, the things they have to do. They don’t tend to ask many questions and they don’t discuss decisions with the consultants. Medical students feel freer to ask and to say “I don’t know” than the team. They have the right to make mistakes and I think the junior doctors don’t, because they share the responsibility of patient care and they fear making mistakes.* Consultant Interview IM H1.

We observed few, if any, instances of explicit feedback in any setting other than surgical theatre. Consultants and senior residents expressed concerns about how and when to give feedback in clinical settings for fear of causing loss of face to the recipient. For example, a consultant described how he used his body language and facial expressions rather than explicit feedback to communicate his concerns or disagreement:

*“The junior doctors are passing you on the results of investigations that they have taken. You are not going to say ‘Why did you do that scan? That’s a ridiculous scan to do, there was no indication to do that scan’. A huge amount of communication is non-verbal. If somebody has done something there is no point in ratting them out in front of everybody else. If you raise an eyebrow you’re doing the same thing as actually saying it. People take up things in different ways and it’s very hard to be explicit in saying “ok this is not working, you’re not showing progression here”.* Consultant interview IM H1.

Similarly, we observed that it was very rare for senior team members to comment explicitly on a junior doctor’s narrative capability. A good case presentation might be acknowledged with a grunt or a nod and by moving the conversation on. If a case presentation was insufficient in some manner, the lead clinician would not communicate concern directly, but might interrupt the presentation by asking a series of clarifying questions or at times physically commandeering the clinical notes.

## Discussion

This study used Goffman’s dramaturgical concepts of impression management and face work to elucidate how teacher and learner roles were co-constructed in relation to the implicit curriculums of clinical teams. This research adds to the literature on learning in teams by describing in much more detail than heretofore (Hawryluck et al. 2002; Lingard et al. 2002a; Hoffman and Donaldson 2004; Balmer et al. 2009; Kennedy et al. 2009a, 2009b; Lewin and Reeves 2011; Hibbert et al. 2018; Vanstone and Grierson 2019) the content of the implicit curriculums that inform socialisation and progression in clinical teams. The study also adds to the literature on faculty development by showing how teacher and learner identities were co-produced in the course of everyday interactions in the micro-organisational contexts of clinical teams. Clinical teachers modelled and embodied, but did not articulate, their standards and expectations. Trainees felt compelled to recognise, interpret, and reproduce the unarticulated norms and standards of their seniors to achieve desired social ends such as recognition, credibility, greater autonomy, and progression. Teachers and trainees colluded in using face work strategies to avoid undermining trainees’ impressions of being capable team members. Worryingly, from a faculty development perspective, lack of clarity about standards and expectations coupled with face work to avoid embarrassment diminished teachers’ and learners’ ability to exploit opportunities for learning and change.

The idiosyncratic and implicit nature of clinical teachers’ standards, judgements and expectations presents challenges for all clinical learners, not just graduate trainees. Ott et al. (2018) for example, found that opportunities for dialogue and feedback were missed during surgical procedures because surgical trainers did not articulate their concerns and judgements occasioned by trainee hesitation. In observational studies of undergraduate clerkships, researchers found that students felt obliged to discover the implicit expectations of clinical teachers in every clinical placement, to optimise their educational opportunities and to achieve better evaluation outcomes.(Han et al. 2015; Vanstone and Grierson 2019). It can be argued that this is simply learning how to play the political game within clinical teams (Vanstone and Grierson 2019), but embodying the realpolitik of a hierarchical team can have negative consequences. For example, in an interview-based study, Patel et al. (2018) found that surgical trainees used a variety of strategies to fake knowing and capability in order to gain credibility and autonomy, but sometimes did so to the detriment of patient safety. The problem of unarticulated standards, “faking it”, and the face work collusion that we found in this study, present important challenges for faculty developers because it shows that the role of clinical teacher is situationally contingent and relational. It follows that faculty developers should avoid imposing educational solutions to rectify the problems of clinical education and should instead collaborate with clinical teachers to develop interventions that maintain the efficiency and effectiveness of clinical teams, whilst mitigating the sometimes negative effects of impression management and face work on clinical learning.

The strategies of impression management and face work that we observed occurred in hierarchical teams characterised by asymmetries of power. Whilst Goffman is sometimes criticised for not accounting for the normative effects of power on impression management (Moore 2017), there are useful definitions of power that can help to explain what was happening in clinical teams. For example, power can be defined as the capacity to “get others to think, feel or act the way we want them to” (Rees et al. 2013). Using this definition, we can see how a relative lack of power, such as that experienced by participants lower down the hierarchy of the clinical teams in this study, diminished their agency in being able to influence senior team members’ articulation of their thinking or secure feedback on performance. Foucault (1975) described how power embedded in social structures such as hierarchical clinical teams can mitigate (learner) agency, but he also described how power can act in “capillary” fashion to implant established ways of thinking and acting so that they become taken for granted, and apparently immutable (Rees et al. 2013). Thus, asymmetries of power built into the structure of clinical teams and reinforced capillary fashion through interpersonal communication can be viewed as potential sites of resistance to the suggestions, (for change) of faculty development. The implication for faculty developers is that they should enable clinical teachers to become much more aware of the effects on learning of the inherently asymmetrical relationships that apply in clinical teams, whilst also facilitating learners to become more agentic in negotiating change for the benefit of their training and progression.

### Limitations

This study has provided important insights into how the role of clinical teacher and graduate learner are co-constructed in clinical teams. In so doing we focused on where the majority of clinical learning occurs, i.e. informally in the collaborative delivery of clinical care. We excluded explicit teaching events because they are relatively uncommon occurrences in the course of clinical practice and are associated with conscious and deliberate performances of teacher and learner identities. Similarly, the data selection for this account does not include our observations of how junior team members modelled and supported each other in terms of mastering the implicit curriculum. Whilst this undoubtedly happened, our focus in this research was on the co-construction of informal clinical learning between senior and junior team members to provide insights that might support future workplace based faculty development initiatives. Whilst an ethnographic approach is very appropriate for studying the relationship between cultural context and the realisation of an implicit curriculum between participants in clinical teams, there are also potential distorting effects in the eye of the beholder. We therefore ensured that the lead author kept an up-to-date reflexivity diary that recorded perspectives, biases, and shifting understandings that emerged during the observation and analysis processes. Moreover, the lead author is an insider observer with outsider perspectives. Being a doctor meant that he could understand what was going on, whilst being a general practitioner allowed him to “make strange" hospital settings. Member checking with the participating doctors and interaction with one of the co-authors, a hospital consultant, ensured that biases and misunderstandings were identified and addressed. The interpretation of field note and interview data was further strengthened by corroboration with video and image data.

### Faculty development recommendations

The contextual turn in faculty development practice and scholarship requires faculty developers to grapple with the so-called “dark matter” of workplace contexts (Bates et al. 2018, O’Sullivan and Irby 2011; Steinert 2012; Steinert et al. 2019). Faculty development interventions for clinical teachers should be founded on a deep understanding of the particularities of the social settings in which those changes are to be situated. In other words, faculty developers should consider creating a “contextual curriculum” designed to exploit the situational affordances of clinical workplaces (Bates et al. 2018). To achieve this, it will be necessary for faculty developers to engage in observational work, witnessing teaching in situ rather than prescribing educational solutions for problems that they cannot fully understand. In addition to direct observation, team members can be recruited as witnesses of team culture and practice. We found, as others have done, (Bates et al. 2018) that new entrants to clinical teams such as interns were particularly sensitive to features of the team’s implicit curriculum. New entrants could therefore be regarded as “diviners of team culture” and encouraged to articulate their observations to senior team members and to faculty developers. Similarly, video reflexive approaches could be used to help team members to become much more self-aware about their embedded practices and behaviours (Iedema et al. 2007). There is strong evidence to show that video reflexive technology can lead to beneficial change to health professional culture and practice (Iedema et al. 2015; Gordon et al. 2017).

In addition to faculty development interventions designed for particular workplace settings, there are also more generic interventions that can usefully be applied in a variety of clinical workplaces. For example, there is growing consensus that teacher identity and engagement with faculty development are enhanced if clinical teachers can be facilitated to join workplace based communities of educators (Jippes et al. 2013; Elmberger et al. 2018) where they can share narratives about their work as teachers (van Lankveld et al. 2017a).

### Recommendations for further research

Our study looked at the realisation of teacher and learner roles within the micro organisational contexts of clinical teams whilst others have looked at the phenomenon of teacher identity in relation to macro organisational settings (van Lankveld et al. 2017b). There is now a need for research looking at the complex relationship between the micro organisational learning environments of the clinical team and affordances of the macro organisational structures in which those teams are situated.

## Conclusions

We have endeavoured to show how lead clinicians and graduate trainees co-construct the roles of clinical teacher and learner in the context of hierarchical clinical teams. In so doing we have shown how an implicit curriculum that applies within clinical teams dictates much of what is learned and how it is learned. We argue that asymmetries of power and the practice of face work act to conserve the norms, values and practices of the implicit curriculum and as such reduce opportunities for learning, development and change.

Despite the fact that faculty development is beginning to reframe teacher development as a contextual and relational process, the emphasis has largely been on the structuring effects of institutional norms and values on clinical teacher development rather than the equally important discursive construction of the teacher in the context of clinical teams. We recommend that the contextual turn in faculty development should not only include a careful exploration of the social suggestions of institutional environments, but also the shaping effects of the social forces that apply within clinical teams. In so doing we are confident that many of the problems encountered in previous efforts to develop clinical teachers will be addressed and overcome.

## Appendix 1: The iterative relationship between analytical dilemmas and data collection

One of the earliest recurring patterns that the lead author observed during data collection was the ritualistic group shapes used by clinical team members during ward rounds. For example, it was noted that the clinical team naturally fell into a hierarchical V formation moving down hospital corridors between wards or assembled themselves into a hierarchical C formation around patient bedsides. These recurring shapes were recorded in the lead author’s field notes using written descriptions and drawings. After some weeks the lead author was also able to share some images showing these configurations with his co-authors. These ritualistic group shapes were of great interest to us because despite being a self-evident norm of hospital practice, they had not been written about much in the past. We could see that other authors had written about ritualistic choreographies within clinical teams e.g. (Balmer et al. 2012), but little or nothing about the hierarchical distribution of team members in hospital corridors and at patient bedsides. Whilst fascinated with what these group shapes were doing, we could not see how they helped us to address our main research interest, i.e. the co-construction of teacher and learner roles within clinical teams. Rather, these group configurations appeared to be serving a different purpose. For example, at the bedside, the team typically arranged itself in a C shape with most of the communication with the patient being led by one individual, supported by others with relevant documents, information and affirmations. The purpose of these bedside team distributions appeared to be a front stage collective performance for the patient as audience, designed to communicate team togetherness and competence. They did not appear to be about the practices of teaching or learning. Having explored the issue in ad hoc interviews with team members, the lead author changed the focus of his observation in situ. Rather than focusing on group distributions, the lead author learned that group interactions before and after such ritualistic team distributions around the bedside were more valuable for the purposes of addressing the research question. For example, it was common practice for team members to present a rapid case synopsis to the lead clinician in the moments before the patient encounter. These case presentations proved much more revealing about the co-construction of teacher and learner roles than the fascinating, but ultimately irrelevant group shape formations.

## Appendix 2: Differing researcher perspectives

The three researchers come from different disciplinary and research backgrounds. The lead author is a general practitioner and academic with an interest in the faculty development of clinical teachers. The second author is an educational psychologist with an interest in group learning and teacher development. The third author is a hospital physician and academic with an interest in workplace learning. The differing interests, experience and perspectives of the three authors led to several debates, discussions and enriched the insights emerging from initial data coding to final interpretations. We provide one such example below.

The lead author observed how junior team members were often asked to negotiate access to hospital services for the teams’ patients without any explanation for the rationale underpinning those tasks. On the face of it, this seemed like a surprising finding, but one of the co-authors, a hospital consultant, was able to explain that this was not just particular to the teams observed in this study, but is common practice in hospital-based teams in the UK. The third author, an educational psychologist, contributed the idea of shared mental modelling between members of teams as a way of conceptualising what was happening implicitly between team members in our sample of clinical teams. The mental modelling literature shows how teams operate on the basis of assumptions of common understanding of phenomena between team members, but also have mechanisms for explicit discussion and explanation. This was clearly lacking in many of the interactions witnessed in our observational study. Using the mental modelling idea, we revisited Goffman’s descriptions of how teams collaborate in presenting front and backstage performances. One of the key features of a collective performance is how team members simulate being in the “in the know” in order to carry off a compelling performance for an audience. In our study, it struck us that being “in the know” was not just a means of carrying off convincing collective impressions, but was also a way of creating an individual impression of capability. Being seen to ask a senior team member for an explanation or rationale risked creating a negative impression and was not often used as a strategy. Thus, we came to understand how the imperatives of individual impression management could undermine shared mental modelling in clinical teams.
